# Supplementary material for: Navigating the risks: Stakeholder views on risk-based cervical cancer screening
Source: PLoS One. 2025 Mar 5;20(3):e0317986. doi: 10.1371/journal.pone.0317986 (PMC11882054; doi:10.1371/journal.pone.0317986)
Supplement: S1 File — (DOCX) [file pone.0317986.s001.docx]

**Supplementary content S1. Interview guide.**

Remmel M-L, Suija K et al. Navigating the risks: stakeholder views on risk-based cervical cancer screening.

Semi-structured interview guide to explore the perspectives of policy and service level stakeholders in on the feasibility of risk-based cervical cancer screening.

| **Components** | **Questions** |
| --- | --- |
| **Initial questions** | The interviewer introduces themselves (name, position), confirms the oral consent, and mentions that the conversation will be recorded. They validate that this is acceptable for the interviewee. The interviewer proceeds with introductory questions.   - What is your year of birth? - What is your professional / educational background? (excluding doctors, nurses, midwives) - How long have you been working in your field (e.g., as a midwife)? - What is your position / duties / responsibilities regarding cervical cancer screening? |
| **Background information on risk-based cervical cancer screening**  A slideshow presentation explaining the concept of risk-based cervical cancer will be given to the interviewee. The script of the video is as follows:  “Nearly all cervical cancer cases are caused by the sexually transmitted human papillomavirus (HPV). Cervical cancer can be prevented with vaccination and detected through regular screening. When identified early, it is often treatable. Certain HPV types are more likely to lead to cervical cancer, known as high-risk HPV subtypes.  There is increasing interest in developing a cervical cancer screening system that considers an individual's specific risk for the disease, a method known as risk-based screening.  In Estonia, women aged 30 to 65 are currently invited for cervical cancer screening every five years, which involves testing for the HPV virus that causes cancer. This system, where all women in a specific age group are screened the same way, is called a "one-size-fits-all" approach.  If personal data, such as age, HPV vaccination status, past screening results, pregnancies, and other health conditions, are collected from national registries and used in a risk calculator, it becomes possible to estimate each woman's individual risk of developing cervical cancer. Based on this calculation, women can receive personalized recommendations on how often they should undergo screening.  This personalized screening method is not yet in use in Estonia. To illustrate risk-based screening, here are two examples:  **Example 1:** Laura, 30 years old, was told her risk of cervical cancer is lower than average due to her young age, normal past screenings, and no history of pregnancy. As a result, she is advised to reduce her screening frequency to once every 10 years instead of every five.  **Example 2:** Sirje, 54 years old, was informed her risk is high because she is over 50, has had a positive HPV test with a high-risk type, has experienced changes in cervical cells, and has had three pregnancies, one of which ended in an abortion. The recommendation for her is to increase screening frequency to once a year instead of every five.” | |
| **Affective attitude** | For doctors and midwives  • Let’s imagine for a moment that you are the one who provided Laura with the information about her low risk of cervical cancer, as mentioned in the example. How would you feel when delivering this information?  • What thoughts and feelings arise when you consider that women with a lower risk of cervical cancer are advised to participate in screening less frequently, as described in Laura's example?  • Let’s imagine for a moment that you are the one who provided Sirje with the information about her high risk of cervical cancer, as mentioned in the example. How would you feel when delivering this information?  • What thoughts and feelings arise when you consider that women with a higher risk of cervical cancer are advised to participate in screening more frequently, as described in Sirje's example?  For policymakers and other stakeholders:   - What emotions and thoughts come up when women are told they have a low risk of cervical cancer?   • What thoughts and feelings come up when women with a lower risk of cervical cancer are advised to participate in screening less frequently, as described in Laura's example?  • What thoughts and feelings come up when women are informed about their high risk of cervical cancer?  • What thoughts and feelings come up when women with a higher risk of cervical cancer are advised to participate in screening more frequently, as described in Sirje's example?  For all stakeholders:  • What do you like about the idea of risk-based screening? What do you dislike?  • Do you think women would want to know their personal risk of developing cervical cancer? Why?  • How do you think women would react to being told they have a low risk of cervical cancer and the recommendation to participate in screening less frequently than before? What emotions, fears, or questions might this raise?  • How do you think women would respond to being informed of a high risk of cervical cancer and the recommendation to participate in screening more often than before? What emotions, fears, or questions might this provoke?  • How would you feel if you were involved in making or implementing the decision to transition to risk-based screening. |
| **Burden** | • If our country adopted a risk-based screening system, what additional information or support would women need regarding this approach? (e.g., information about cervical cancer/HPV; assurance that confidentiality issues are addressed)  • Do you think women would want to know why their cervical cancer risk is high or low? Why?  • How should the risk threshold be defined (i.e., what constitutes low and high risk)?  • Who should decide this?  • Who and how should determine the appropriate screening interval for each risk group?  • If the country adopted a risk-based screening system, what additional information or support would you as a professional need regarding this approach?  • What obstacles might hinder informing women and sharing information about the new risk-based screening system? |
| **Ethics** | - Do you think it is fair for each woman to receive a personalized recommendation on how often she should undergo cervical cancer screening? - One could say that risk-based screening would create unequal access to health monitoring for individuals. What do you think of this idea? - What do you think about using women’s personal health data to calculate individual risk? To what extent would this help women decide about participating in screening? - The calculation of personal cervical cancer risk is more accurate when information such as the start of sexual activity, number of sexual partners, and smoking is included. To what extent would a more accurate risk calculation justify including this information? - What do you think about the possibility of women being able to add information themselves into a risk calculator available to them? - Which would be preferred: a less accurate risk calculation without asking for additional information, or asking for additional information and getting a more precise risk calculation? Why? - In rare cases, the calculated risk based on health registry data may be higher than the actual risk (a false positive result). In such case, a woman would be recommended screening (HPV testing) too frequently. In some cases, the opposite may occur and the calculated risk based on health registry data may be too low (a false negative result). In this case, one would participate in screening too infrequently, e.g., once every 10 years, and early changes or cancer may be detected too late. What thoughts and feelings does the potential inaccuracy of risk assessment evoke in you? - Risk-based screening can be highly beneficial for those with high risk, ensuring they are tested frequently and receive timely treatment if needed. However, in most cases, women with a high estimated risk do not develop cervical cancer. In such cases, frequent testing may be seen as excessive intervention and not beneficial to the individual. What thoughts and feelings do these aspects evoke in you? |
|  |  |
| **Intervention coherence** | - To what extent do you feel it is clear to you why risk-based screening might be beneficial? - What do you think—how accurately can risk be assessed using information from national health data registries? - Do you agree that women with a higher risk of developing cervical cancer should be tested more frequently? Why? - Do you agree that women with a low risk of cervical cancer should be tested less frequently? Why? |
| **Opportunity costs** | - How would the introduction of risk-based screening affect your responsibilities in screening?   For midviwes and doctors:   - What additional information, training, or other resources would you need regarding risk-based screening?   For other stakeholders:   - What information and additional resources would you need (e.g., human resources, technological solutions, etc.) to manage and implement a risk-based screening strategy? - What could facilitate the process of developing a risk-based screening strategy? |
|  |  |
| **Perceived effectiveness** | • What do you think—how much would risk-based screening, compared to age-based screening, help to involve more women in screening?  • To what extent do you agree that risk-based screening would prevent more cases of cervical cancer? How?  • Which method would you prefer— the current "invitation for everyone every 5 years" or individual risk-based screening? Why?  • What do you think are the most important indicators for measuring the effectiveness of the cervical cancer screening program? |
| **Self-efficacy** | - What potential obstacles do you think might arise in your work when implementing, organizing, or managing risk-based screening? - What would help to implement and promote risk-based screening in your professional position? |
| **Conclusion** | - Is there anything else you would like to address regarding risk-based screening that has not been mentioned in the interview?   Thank you very much for the interview. |
