# Supplementary material for: Navigating the risks: Stakeholder views on risk-based cervical cancer screening
Source: PLoS One. 2025 Mar 5;20(3):e0317986. doi: 10.1371/journal.pone.0317986 (PMC11882054; doi:10.1371/journal.pone.0317986)
Supplement: Attachment — Submitted filename: Response to Reviewers.docx [file pone.0317986.s003.docx]

Dear Editor Dr. Clement A. Adebamowo,

I would like to thank you for the opportunity to submit a revised manuscript titled “Navigating the risks: stakeholder views on risk-based cervical cancer screening.” to *PLOS ONE.*

We appreciate the time and effort that you and the reviewers have dedicated to providing your thoughtful and constructive feedback on our manuscript.

We believe that we have addressed all the concerns raised in the review and that the manuscript has been strengthened as a result.

Our responses to the review comments are presented below. All changes made in the manuscript are highlighted as track changes.

Thank you for your consideration of this manuscript.

We look forward to hearing from you regarding our submission and are ready to address any further questions and comments you may have.

Best regards,

Maali-Liina Remmel

| Editorial comments | |
| --- | --- |
| 1. Please ensure that your manuscript meets PLOS ONE's style requirements, including those for file naming. | The manuscript has been reviewed and corrected as per PLOS ONE's style requirements, including those related to file naming. |
| 2. Could you please provide further details on why your study is exempt from the need for approval and confirmation from your institutional review board or research ethics committee (e.g., in the form of a letter or email correspondence) that ethics review was not necessary for this study? Please include a copy of the correspondence as an "Other" file | We appreciate the opportunity to address the editor's request for further details regarding the ethical considerations of our study. Ethical approval was not sought as the study qualifies for exemption according to the applicable ethics committee regulations and Estonian legislation.^1,2^  Specifically, the study:  (i) did not collect medical data;  (ii) did not involve patients;  (iii) focused on professional knowledge within participants public roles;  (iv) did not involve sensitive data;  and  (v) posed minimal risk to participants.  The study complied with the ethical guidelines set forth in the Belmont Report. To ensure participant confidentiality, all data were de-identified. Informed consent was obtained from all participants after they were fully briefed on the study's purpose, procedures, their rights to withdraw, potential risks or discomforts, possible benefits, confidentiality safeguards, and contact details for addressing any questions regarding the research.  While the University of Tartu's ethics committee does not provide formal confirmations of exemption^3^, similar studies typically do not require ethical approval in other jurisdictions, as illustrated by the UK's NHS Health Research Authority and NIH Grant/funding decision tools^4^ (results attached as an Other file).  Several categories of research are typically exempt from ethics review. A recent review of national guidance in developed countries, including those from Australia, the United Kingdom, the United States, and the Netherlands, identified nine such categories. One common exemption is "research with staff in their professional role," which applies to our study.^5^  We believe this clarifies the ethical considerations of our study and its compliance with relevant regulations  E. ^1^Estonian Personal Data Protection Act. Available from: https://www.riigiteataja.ee/en/eli/ee/Riigikogu/act/507112023002/consolide (Accessed 12.12.24)  21. ^2^University of Tartu Guide for data protection in  Research. Available from: https://wiki.ut.ee/pages/viewpage.action?pageId=119967472 (Accessed 12.12.24)  ^3^Research Ethics Committee of the University of Tartu. Available from:  https://ut.ee/en/node/113848. Accessed 12.12.24  ^4^HRA decision tool. Available from:  https://www.hra-decisiontools.org.uk/ethics/. Accessed 09.12.24  ^5^ Scott AM, Kolstoe S, Ploem MCC, Hammatt Z, Glasziou P. Exempting low-risk health and medical research from ethics reviews: comparing Australia, the United Kingdom, the United States and the Netherlands. Health Res Policy Syst. 2020 Jan 28;18(1):11. doi: 10.1186/s12961-019-0520-4. |
| 3. Please remove any funding-related text from the manuscript and let us know how you would like to update your Funding Statement. | This text has been removed and no updates are required for the current Funding Statement. |
| 4. We note that you have indicated that there are restrictions to data sharing for this study. For studies involving human research participant data or other sensitive data, we encourage authors to share de-identified or anonymized data. However, when data cannot be publicly shared for ethical reasons, we allow authors to make their data sets available upon request. For information on unacceptable data access restrictions, please see <http://journals.plos.org/plosone/s/data-availability#loc-unacceptable-data-access-restrictions>.  Before we proceed with your manuscript, please address the following prompts:  a) If there are ethical or legal restrictions on sharing a de-identified data set, please explain them in detail (e.g., data contain potentially identifying or sensitive patient information, data are owned by a third-party organization, etc.) and who has imposed them (e.g., a Research Ethics Committee or Institutional Review Board, etc.). Please also provide contact information for a data access committee, ethics committee, or other institutional body to which data requests may be sent.  b) If there are no restrictions, please upload the minimal anonymized data set necessary to replicate your study findings to a stable, public repository and provide us with the relevant URLs, DOIs, or accession numbers. Please see <http://www.bmj.com/content/340/bmj.c181.long> for guidelines on how to de-identify and prepare clinical data for publication. For a list of recommended repositories, please see <https://journals.plos.org/plosone/s/recommended-repositories>. You also have the option of uploading the data as Supporting Information files, but we would recommend depositing data directly to a data repository if possible.  Please update your Data Availability statement in the submission form accordingly. | We acknowledge the importance of addressing this matter.   a) Even if deanonymized, indirect identifiers may risk the identification of individuals participating in this study. Therefore, to protect their confidentiality, the data will not be publicly shared. However, deanonymized data could be shared upon reasonable request. The data is in Estonian language. Requests regarding data should be sent to [eetikakomitee@ut.ee](mailto:eetikakomitee@ut.ee) (Tartu University Research Ethics Committee). |
| 5. Please review your reference list to ensure that it is complete and correct. If you have cited papers that have been retracted, please include the rationale for doing so in the manuscript text, or remove these references and replace them with relevant current references. Any changes to the reference list should be mentioned in the rebuttal letter that accompanies your revised manuscript. If you need to cite a retracted article, indicate the article’s retracted status in the References list and also include a citation and full reference for the retraction notice. | We have carefully reviewed the reference list to ensure it is complete and accurate. |
| Reviewer #1 | |
| Abstract  Line 40: The abbreviation ‘RB’ has not been previously introduced, please include this on line 30 where the term risk-based is first used. | The abbreviation ‘RB’ has now been introduced where the term "risk-based" is first mentioned. We appreciate your attention to detail. |
| Introduction  Lines 81-83: The focus of this study appears to be acceptability but the authors state that they are aiming to assess feasibility. Acceptability and feasibility are two distinct concepts, and I would avoid using these as synonyms. Based on the rest of the manuscript it seems that the authors are in fact aiming to assess acceptability and that, in the case of stakeholders, feasibility is a component of that. | We appreciate the reviewer’s insightful comment and fully agree that acceptability and feasibility are distinct concepts. Upon reflection, we recognize that the primary focus of our study is indeed on assessing the acceptability of risk-based cervical cancer screening (RB CCS) programs, with feasibility being considered as a component within the broader concept of acceptability, particularly from the stakeholders' perspectives. We have revised the manuscript accordingly. Thank you for bringing this to our attention |
| Methods  Lines 89-92: Please explain why you chose the TFA to develop your interview guide instead of the CFIR. I am curious as to why both of these frameworks have been used instead of just one, typically if a framework is used to inform the study design then the same framework is used in analysis. | Thank you for this insightful question. We appreciate the opportunity to clarify our choice of frameworks. While it's true that a single framework often guides both design and analysis, our study's needs led us to utilize both the TFA and CFIR. As we understand, TFA allows in-depth understanding of acceptability. Our primary focus was to explore the acceptability of the risk-based cancer screening among relevant stakeholders, with its specific focus on individual perceptions and attitudes, provided an opportunity to assess the factors influencing their willingness to adopt this type of screening. It allowed us to design the interview guide with questions directly addressing the seven constructs of the TFA (affective attitude, burden, ethicality, etc.), providing rich qualitative data on user acceptance. While understanding acceptability was crucial, we also recognized the importance of the broader implementation context.  The CFIR helped us capture this wider perspective by considering factors beyond individual acceptance. During analysis, the CFIR allowed us to examine how elements like the inner setting (e.g., existing healthcare infrastructure), outer setting (e.g., policies in the host countries), and implementation process might interact with and influence the acceptability of the risk-based cancer screening.  Furthermore, the decision to incorporate the CFIR as a complementary framework was also data-driven— as the interviews progressed, it became evident that participants frequently brought up factors related to the broader implementation context. Recognizing this, we decided to integrate the CFIR into our analysis to provide a more comprehensive structuring and deeper exploration of the data.  We view the TFA and CFIR as complementary rather than competing frameworks and our approach allowed us to balance the in-depth exploration of individual acceptability with a nuanced examination of the wider implementation landscape, leveraging the strengths of both frameworks.  Clarifications were added to the manuscript. |
| Methods  Line 158: The data analysis section is clear and well-explained. | Thank you for your positive feedback on the data analysis section. |
| Methods  Line 174: Again, it would be beneficial to explain why CFIR was chosen at the analysis stage in favour of the TFA. | Clarifications were added to the manuscript. |
| Results  Lines 329-331: The comparison between different types of stakeholders, in this case midwives and gynaecologists, is very interesting. If such differences were observed elsewhere in the analysis, I would encourage drawing these out explicitly. | Thank you for highlighting this point. Beyond the differences explicitly noted in the manuscript, no other clearly distinguishable differences were observed among stakeholder groups during the analysis. |
| Discussion  Overall, this is an extremely well-written discussion which contextualises your findings nicely. Lines 360-363: This is an interesting and novel contribution to the literature. | Thank you very much for your kind feedback on our discussion section. |
| Reviewer #2 | |
| 1. Line 68: Please add some references to examples (ref 32,33 etc) of existing research. | Thank you for your suggestion. We have now added relevant references to examples of existing research to support and contextualize the statement. |
| 2. The term ‘stakeholder’. The authors rightly acknowledge that community/patients may not be seen as a priority stakeholder group in the study’s context. However, by definition and in other contexts they are. I think ‘Stakeholder’ is acceptable for the Title but suggest clarifying ‘which stakeholders’ earlier in the paper and within Figure 1 to avoid confusion, e.g., Line 82: add ‘healthcare service and policy level stakeholders’ Figure 1: Suggest make title clearer or highlight that concepts related to “Individuals-women” are from the perspective of healthcare policy and service level providers, so this figure can stand alone. | Thank you for this thoughtful feedback. We agree with your suggestion to clarify the term "stakeholder" earlier in the paper and in Figure 1 to avoid confusion. We clarified "healthcare service and policy-level stakeholders" in line 82 and revised the title of Figure 1 and added a clarification to the “Individuals-women” category to emphasize that the concepts reflect the perspectives of healthcare policy and service-level providers. |
| 3. Line 194-195: Check format of SD is correct i.e., suggest (SD=8.6) | Thank you for pointing this out. We have revised the format. |
| 4. Line 266: What is the relevance of digital literacy in this context? Was there a link to inequalities related to digital literacy in your tables? Please add a sentence for clarity. Consider also in the Discussion (Line 402). | Thank you for your comment. Digital literacy is relevant in this context because it was highlighted by stakeholders as a potential barrier to equitable participation in risk-based screening programs. Specifically, concerns were raised about whether all women would have equal ability to navigate digital tools, such as using an online risk calculator, which could lead to disparities in participation. We have added a sentence in the Results section to clarify this point and in the Discussion. |
| 5. Line 282: Headings in Table 3 (‘Inner setting and Outer setting’) are back-to-front. Please review and correct. | Thank you for pointing this out. We have reviewed the headings in Table 3 and corrected them to ensure they are appropriately assigned. We appreciate your careful attention to detail. |
| 6. Figure 1: This looks attractive and potentially useful for researchers and planners. Suggest but not required to combine’ Innovation’ heading/graphic and concepts as one box, and ‘Implementation’ heading/graphic and concepts similarly to reduce complexity. See comments above re: title and women | Thank you for your positive feedback on Figure 1. We appreciate your suggestion to combine the ‘Innovation’ and ‘Implementation’ headings/graphics with their respective concepts to reduce complexity. Based on your recommendation, we have made the suggested modifications to simplify the figure while retaining its clarity. Additionally, we have addressed the comments regarding the title and women above. |
